# CLAVATA1 controls distinct signaling outputs that buffer shoot stem cell proliferation through a two-step transcriptional compensation loop

**DOI:** 10.1371/journal.pgen.1006681

**Published:** 2017-03-29

**Authors:** Zachary L. Nimchuk

**Affiliations:** 1 Department of Biology, University of North Carolina at Chapel Hill, Chapel Hill, NC, United States of America; 2 Curriculum in Genetics and Molecular Biology, University of North Carolina at Chapel Hill, Chapel Hill, NC, United States of America; University of California Berkeley, UNITED STATES

## Abstract

The regulation of stem cell proliferation in plants is controlled by intercellular signaling pathways driven by the diffusible CLAVATA3 (CLV3p) peptide. CLV3p perception is thought to be mediated by an overlapping array of receptors in the stem cell niche including the transmembrane receptor kinase CLV1, Receptor-Like Protein Kinase 2 (RPK2), and a dimer of the receptor-like protein CLV2 and the CORYNE (CRN) pseudokinase. Mutations in these receptors have qualitatively similar effects on stem cell function but it is unclear if this represents common or divergent signaling outputs. Previous work in heterologous systems has suggested that CLV1, RPK2 and CLV2/CRN could form higher order complexes but it is also unclear what relevance these putative complexes have to *in vivo* receptor functions. Here I use the *in vivo* regulation of a specific transcriptional target of CLV1 signaling in Arabidopsis to demonstrate that, despite the phenotypic similarities between the different receptor mutants, CLV1 controls distinct signaling outputs in living stem cell niches independent of other receptors. This regulation is separable from stem cell proliferation driven by *WUSCHEL*, a proposed common transcriptional target of CLV3p signaling. In addition, in the absence of *CLV1*, CLV1-related receptor kinases are ectopically expressed but also buffer stem cell proliferation through the auto-repression of their own expression. Collectively these data reveal a unique *in vivo* role for CLV1 separable from other stem cell receptors and provides a framework for dissecting the signaling outputs in stem cell regulation.

## Author summary

The proliferation of plant stem cells in above ground tissues is controlled by a suite of receptors in response to the CLAVATA3 peptide ligand. Receptor signaling in response to CLAVATA3 prevents over-proliferation of stem cells. It is unclear what the functional relationship is between the proposed CLAVATA3 receptors or if they impact common signaling outputs. Here I demonstrate that CLAVATA1 signals independently of the other receptors kinases to control distinct transcriptional outputs independent of stem cell proliferation. Stem cell proliferation is buffered by a two-step mechanism which transcriptionally regulates receptor levels in the stem cell niche. This mechanism helps explain the strict control of stem cell proliferation and could provide new avenues for improving plant growth.

## Introduction

Co-operative receptor kinase function is a common feature in both animal and plant signaling systems. Receptor kinase mutants are frequently genetically additive in plants but the molecular mechanisms underlying this effect are often different. For instance, double mutants between the *EF-TU RECEPTOR* and *FLAGELLIN SENSITIVE2* receptor kinases display enhanced susceptibility to bacterial infection above each single mutant [1], reflecting differences in pathogen derived ligands, followed by quantitative activation of common downstream outputs. On the other hand, additive genetic interactions among mutants in *SOMATIC EMBRYOGENESIS RECEPTOR-LIKE KINASE* family co-receptor kinases in response to specific ligands reflect quantitative redundancy as co-receptors [2]. Dissecting the molecular basis of redundancy in gene families in plants is often also complicated by unequal contribution from distinct genes and often requires *in vivo* analysis of signaling outputs or component interactions [3]. Balanced stem cell production in shoot (SAM) and floral meristems (FMs) is mediated by cell-to-cell signaling pathways initiated by the CLAVATA3 (CLV3) peptide ligand, a founding member of the CLE family of peptides [4]. Mutations in *CLV3* lead to excess accumulation of stem cells in both SAM and FMs [5]. CLV3p is thought to be perceived by a series of overlapping receptor kinases which signal to dampen stem cell production. To date, much of the analysis of these receptor-ligand mutants has been at the gross morphological level. Mutations in the different proposed receptors vary considerably in their strength and genetic interactions. It is unclear if the morphological similarities, strength differences, or genetic interactions are due to co-operative cross talk, convergence on a common signaling output, or divergent pathways.

CLV3 is secreted from stem cells in the growing tip of the meristem, proteolytically processed and modified to a 13 amino acid diffusible glycopeptide (CLV3p) which diffuses broadly throughout the SAM [6–10]. Genetically, CLV3p perception is mediated by the transmembrane receptor kinase CLV1 [11–13], but also by a heterodimer of the receptor like protein CLV2 and the transmembrane pseudokinase CORYNE (CRN, [14–17]). Additionally, the receptor kinase RECEPTOR-LIKE PROTEIN KINASE 2 (RPK2) may also function in CLV3p perception [18]. All four mutants are resistant to exogenous CLV3p treatment to different degrees suggesting that they act as receptors for CLV3p *in vivo*. Previous data using overexpression in differentiated tobacco leaf cells has suggested that CLV1 could form higher order complexes with CLV2/CRN leading to the hypothesis that they may signal co-operatively in the SAM [19–22]. However conflicting results have been obtained by different groups and it is not clear if such complexes form in SAM tissues, or when receptors are expressed at endogenous levels in the appropriate cell types. While CLV3p has been reproducibly demonstrated to bind the CLV1 ectodomain [12, 23], differing results have been obtained for the ability of CLV2 to bind CLV3p [20, 23]. In addition, these studies have not tested for potential co-receptor binding of CLV3p. As such it is unclear how, or if, these receptors control stem cell proliferation co-operatively *in vivo*.

Strong alleles of *clv1*, such as *clv1-4* and *clv1-8*, contain missense mutations in the LRR domain of CLV1 [11]. The residues effected in these mutants are highly conserved among related LRR-RKs and structural and biochemical studies with PXY/TDIF receptor ligand pair have shown that these residues direct ligand binding [24–28]. In contrast, *clv1* null mutants are significantly weaker [29]. The weak stem cell defects in *clv1* null mutant plants can partially be explained by the compensatory up-regulation of *CLV1*-related receptor kinases *BARELY ANY MERISTEM1*(*BAM1*), *BAM2* and *BAM3* [28]. In wild type plants, *BAM* receptor expression in the center of the SAM, where *CLV1* is highly expressed, is undetectable. Consistent with this observation, triple mutants in *bam1 bam2 bam3* have no defects in stem cell regulation on their own. In *clv1* mutant SAMs *BAM* receptors are ectopically expressed in the center of the SAM and partially compensate for *clv1*. However it is unclear why null *clv1* alleles are only weakly compensated by ectopic *BAM* expression. It is also unclear why strong *clv1* alleles are phenotypically more severe. Previous work has suggested that strong clv1 mutant receptors may interfere with CRN/CLV2 signaling [16]. Alternatively, strong clv1 mutant receptors have been suggested to interfere with BAM signaling [30]. It is not clear how this relates to the feedback regulation of *BAM* expression by CLV1.

CLV1, and the other putative CLV3p receptors, are proposed to negatively regulate *WUSCHEL* (*WUS*) expression in the center of the SAM [13, 31]. WUS is a homeodomain transcription factor and de-repression of *WUS* in *clv3* mutants is thought to drive excess stem cell proliferation [31, 32]. Despite this, the expression of *WUS* is robust and co-incident with *CLV1* in wild type plants in the center of the SAM and *WUS* levels do not change dramatically at the cellular levels in response to loss of *CLV1* signaling [11, 28, 32, 33]. Unlike *WUS*, CLV3p-CLV1 signaling fully represses *BAM* expression in the center of the SAM in wild type plants [28]. Plants expressing up to 300 fold higher levels of *CLV3* have a wild type appearance, suggesting that repression of *WUS* is most effective outside of the physiological range of CLV3p concentration [34]. Interestingly, expression of *CLV1* from the *WUS* promoter is necessary and sufficient to fully complement both *clv1* null mutants and *clv1 bam1 bam2 bam3* quadruple null mutants back to wild type levels of stem cell regulation [28]. As such, *CLV1* operates exclusively in *WUS* expressing cells of the SAM, despite *WUS* being a target for transcriptional repression. It is not clear where in the SAM other proposed CLV3p receptors function or if WUS-mediated cell proliferation is linked to *BAM* transcriptional regulation by CLV1 *in vivo*.

Here I use quantitative genetics, and the highly specific transcriptional repression of *BAM3* by CLV1 to demonstrate that CLV1 signals independent of CRN, CLV2 and RPK2 in response to CLV3p *in vivo*. In *clv1* null mutants, ectopic BAM receptors compensate for CLV1 but also act in an additional feedback loop to dampen their own expression in the SAM and buffer stem cell proliferation. Strong alleles of clv1 specifically interfere with this process and have no impact on CLV2/CRN function. Despite their proposed ability to repress *WUS*, CRN/CLV2 function exclusively in *WUS* expressing cells of the SAM like *CLV1*. Consistent with this, *WUS*-induced stem cell proliferation is genetically separable from *BAM3* regulation by *CLV1*. My data demonstrate that despite the qualitative phenotypic similarities, CLV1 signaling outputs diverge from other receptors, and from *WUS*, and support a model in which CLV1 is functionally independent of other proposed receptors *in vivo*.

## Results

### Generation of a *crn* null mutant

**Fig 1:**
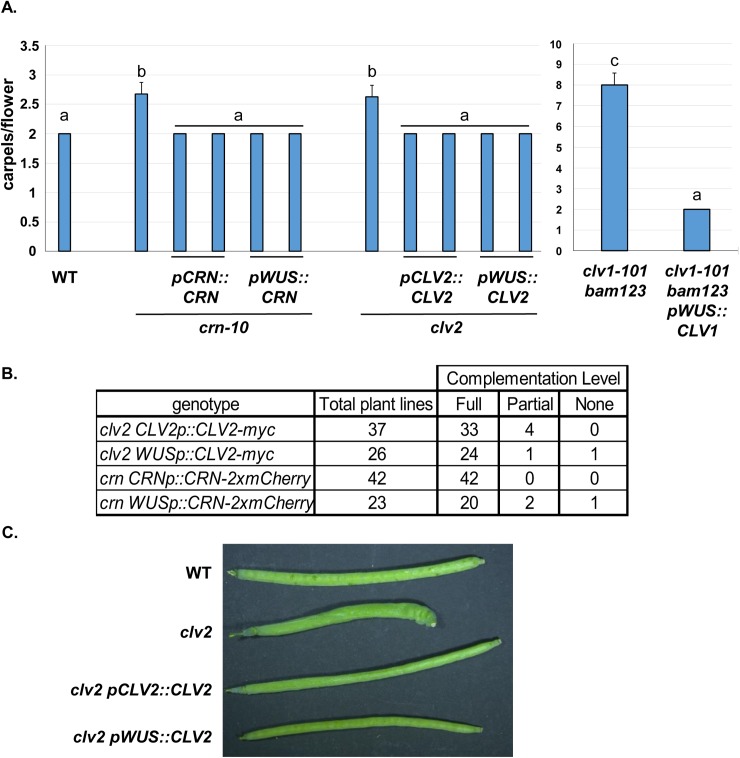
CLV3p receptors function in *WUS*-expressing cells is sufficient to control stem cell proliferation. (A) CRN, CLV2 and CLV1 control stem cell production in WUS expressing cells. Previous work demonstrated that CLV1 function in *WUS* expressing cells is necessary and sufficient for stem cell regulation in vegetative and floral meristems in *clv1-101* null mutants and *clv1-101 bam1 bam2 bam3* quadruple mutant plant (*clv1-101 bam123*, [28]). Like CLV1, *crn-10* and *clv2* null mutants were complemented fully by expression of CRN-2xmCherry or CLV2-Myc fusion proteins from either their native promoter or the *WUS* promoter. Y-axis, mean number of carpels per flower for different genotypes, with two independent transgenic lines per genotype displayed. Error bars represent the calculated 95% C.I. N = 40 flowers counted per plant. Letters above bars indicate significance groups as determined by Tukey HSD test/ANOVA. (B) Complementation rates for *clv2* and *crn* transgenic lines. Full complementation was defined as no flower containing more than 2 carpels as in wild type (WT). (C) Example of full complementation in carpels from *clv2* transgenic lines. Similar results obtained with *crn-10* complementation.

In order to determine the functional relationship between the proposed CLV3p receptors I used previously published null alleles in *CLV1*, *CLV2*, *BAM1*, *BAM2* and *BAM3* in the Col-0 background [28, 35]. To date there are no null EMS generated alleles of *CRN* in any ecotype and no T-DNA insertions in the *CRN* coding sequence. I therefore used Cas9 to target *CRN* and create a null mutant. I created a gRNA targeting the signal sequence encoding region of the *CRN* gene and used the *pCUT* series of Cas9 vectors to create indels in the *CRN* gene in the Col-0 ecotype [36]. One of these, hereby referred to as *crn-10*, introduced a single thymine base between bases 20 and 21 in the *CRN* CDS. The mutation introduces a frameshift in the protein after amino acid 6 in the 33 amino acid signal sequence, leading to three in-frame stop codons four amino acids downstream. No other in frame methionine residues are present in, or before, the predicted CRN transmembrane sequence. Thus, the *crn-10* allele retains 6 amino acids out of the original 402 in CRN and creates an early stop codon in the CRN signal sequence and deletes all downstream domains of CRN. *crn-10* was segregated away from the Cas9 transgene for all subsequent work. *crn-10* plants are qualitatively similar to other published *crn* alleles [16], with no new phenotypes noted. Wild type FMs give rise to stem cell populations that support stereotypical numbers of floral organs, culminating in the production of two central fused carpels. In *clv* mutant FMs, the enhanced rate of stem cell production results in increases in floral organ numbers, providing a quantitative measure of stem cell defects [29]. *crn-10* plants displayed increased carpel number with a strength comparable to existing *clv2* null alleles in Col-0 (Fig 1A). As such *crn-10* behaves similarly to recessive non-null EMS alleles of *crn* such as *crn-1* in the La-*er* ecotype [16]. *crn-10* was fully complemented by a *CRN-2xmCherry* fusion protein expressed from the endogenous *CRN* promoter (44/44 lines, S1A Fig) as expected [17]. Previous work aimed at testing the hypothesis that CRN encoded a pseudokinase demonstrated that expression of a CRN_K146E_ mutant protein, which further mutates the conserved active site lysine in CRN, fully complements *crn-1* when expressed from the native *CRN* promoter [22]. At the time *crn-1* was the only allele available and encodes a protein with a missense mutation in the CRN transmembrane domain (G70E) [16]. It is formally possible that if CRN homodimerized, the crn-1 and CRN_K146E_ proteins could cross complement. I therefore transformed *crn-10* with a *pCRN*::*CRN*_*K146E*_-2xmCherry transgene. Again I observed full complementation supporting the previous designation of CRN as a pseudokinase (26/26 lines, S1A Fig). Recent work has suggested that phosphorylation of CRN at serine 156 is important for function and that S156A substitutions fail to complement *crn-1* when expressed from the *35S* promoter [37]. In contrast, expression of either a S156A or a S156D *CRN* variant fully complemented the *crn-10* null mutant when expressed from the *CRN* native promoter (26/27 and 24/24 line displaying full complementation for the S156A and S156D CRN variants respectively, S1A Fig). The reason for the different complementation results are unknown but could reflect either the use of different *crn* mutant plants or different transgene promoters in the complementation experiments.

### *CLV1*, *CRN* and *CLV2* control stem cell regulation exclusively in *WUS* expressing cells of the SAM

I previously demonstrated that *CLV1* function in *WUS* expressing cells is necessary and sufficient for stem cell regulation [28]. Both *CLV2* and *CRN* are expressed broadly in inflorescence tissue (see S1B Fig, [15, 16]) but it is not clear if they function in *WUS* expressing cells of the SAM like *CLV1*. I therefore tested the ability of CLV2-myc and CRN-2xmCherry fusion proteins to complement their respective null mutants when expressed from either their native promoters or the *WUS* promoter. Lik*e CRN*, expression of *CLV2-myc* from the native *CLV2* promoter in *clv2* null mutant plants (*rpl10-1*, [35]) fully complemented stem cell defects in the majority of lines (Fig 1). Both *CRN* and *CLV2* expression from the *WUS* promoter also fully complemented their respective null mutant plants in the majority of T1 lines (Fig 1). Fully complemented lines contained no flowers with more than two carpels, a level of complementation equivalent to complementation of the *clv1 bam1 bam2 bam3* quadruple provided by the *pWUS*::*CLV1-2xGFP* transgene as previously reported [28]. Collectively these data indicate that like CLV1, CRN and CLV2 function exclusively in *WUS* expressing cells of the SAM and FM.

### CRN, CLV2, and RPK2 are dispensable for CLV1-mediated repression of *BAM* receptors

**Fig 2:**
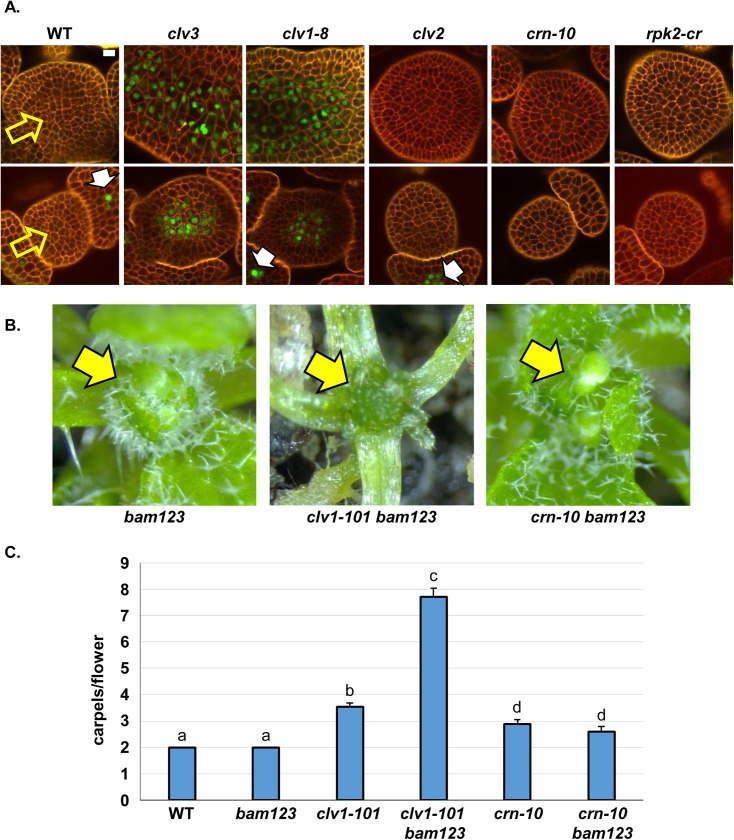
CLV1 signals independent of other proposed CLV3p receptors to control *BAM3* expression. (A) CRN and CLV2 are dispensable for CLV3-CLV1 mediated *BAM3* repression. *BAM3*::*Ypet-N7* signal is off in the center of wild type (WT) SAMs (top row, yellow arrow) or FMs (bottom row, yellow arrow), but is de-repressed specifically in *clv3* or *clv1-8* plants. Note the phloem derived *BAM3*::*Ypet-N7* signal in developing sepals (white arrows) in some imaging planes. Phloem nuclei in other images are not in image plane but still express Ypet-N7. Tissues were stained with propidium iodide (red) to visualize cell walls. SAMs and FMs were imaged by CSLM and each image slice represents the L5 cell layer as determined by counting cell layers from the surface of the SAM or FM. *pBAM3*::*Ypet-N7* fluorescence signal was false colored green to provide better contrast with the red channel. For each genotype, 10 SAMs or FMs were imaged and experiments were repeated three times. White bars, 10 μM. All subpanels are at the same image magnification. See methods for details on *rpk2-cr* null allele. (B) CRN is not required for CLV1 mediated repression of *BAM* receptors. *clv1-101 bam1 bam2 bam3* (*clv1-101 bam123*) quadruple mutant plants display uncontrolled vegetative SAM proliferation (yellow arrow), not seen in *bam123* triple mutant plants, *crn-10 bam123* quadruple mutant plants (yellow arrows). (C) CRN is not required for CLV1 mediated repression of *BAM* receptors in floral meristems. Y-axis, mean carpels number per flower for different genotypes. Ectopic *BAM* expression masks full loss of *clv1* in *clv1-101* null mutants, and *clv1-101* null mutants are strongly enhanced by simultaneous mutation of *bam1 bam2 bam3* (*bam123)*, In contrast, *crn-10* null mutations are strictly additive with *bam123* triple mutants and resemble *crn-10* alone. N = 60, experiment repeated twice. Error bars represent the calculated 95% C.I. Letters above bars indicate significance groups as determined by Tukey HSD test/ANOVA.

My data demonstrate that *CRN*, *CLV2* and *CLV1* all function exclusively in *WUS*-expressing cells in the center of the meristem, however, this observation does not address if they act together at the biochemical level to converge on similar signaling outputs. In wild type plants *CLV1* represses the expression of the related *BAM* receptors in the center of the SAM in response to CLV3p [28]. I therefore asked if CRN and CLV2 participated in CLV1-mediated repression of *BAM3* in the SAM center. For simplicity, *BAM3* expression was analyzed since *BAM1*, *BAM2* and *BAM3* are all targets of CLV1 in the SAM center, but *BAM1* and *BAM2* display expression in the SAM epidermis and floral primorida which is *clv1*-independant [28]. I introgressed the previously characterized *pBAM3::Ypet-N7* transgenic line [28] from Col-0 into the null alleles of *clv2* and *crn*, and isolated homozygous transgenic lines in each mutant background. For *rpk2*, a CRISPR null (*rpk2-cr*) was generated directly in the homozygous *pBAM3::Ypet-N7* wild type transgenic line, and segregated away from the Cas9 transgene for analysis (see Materials and Methods). The *pBAM3::Ypet-N7* reporter generates a tandem Ypet fusion protein that is targeted to the nucleus. I then compared the expression of the *BAM3* reporter in the L3-L5 cells of the SAM center (see S2 Fig for image calibration). *CLV1* is expressed strongly in L3-L5 cells in wild type, *clv3*, and *clv1*-8 meristems and expression in these cells is sufficient to account for all stem cell regulation by CLV1 [25]. Consistent with previous imaging, *BAM3* reporter expression was undetectable in the center of the SAM in wild type plants, but was robustly detectable in *clv3* mutants and strong alleles of *clv1* (Fig 2A). Similar results were found in FMs (Fig 2A), consistent with CLV3p-CLV1 repression of *BAM3* expression in both meristems [28]. In contrast, *BAM3* was not expressed in the center of SAMs or FMs in either *clv2* or *crn* mutants (Fig 2A, see S2 Fig for imaging calibration). *BAM3* is expressed in phloem lineage cells independent of CLV3-CLV1 signaling [28, 38]. In all plants examined, *BAM3* reporter expression was observed in the phloem linage cells of the vasculature outside of the SAM, consistent with previous work [28], demonstrating that lack of signal in the SAM was not due to reporter silencing in any one line (for example see S4 Fig). Similarly, no ectopic expression of *BAM*3 was observed in the L3-L5 cells from SAMs or FMs of *rpk2* null mutant plants Fig 2A, see Materials and Methods for construction of *rpk2* null mutant) [18]. These data demonstrate that CLV1 signals to repress *BAM3* in response to CLV3p independent of CLV2, CRN, and RPK2.

I sought to test this observation genetically by creating higher order receptor mutants. Owing to their repression by CLV1 in the center of the SAM, BAM receptors do not normally participate in stem cell regulation leading to an invariant two carpels per flower in *bam1 bam2 bam3* triple mutants as in wild type Col-0 plants. However, when ectopically expressed in the center of FMs and SAMs in *clv1-101* null mutants, *BAM* receptors partially compensate for the lack of *CLV1* and correspondingly *clv1-101 bam1 bam2 bam*3 mutants greatly enhance carpel numbers of *clv1-101* null mutants and display massive SAM over-proliferation during vegetative growth (Fig 2B and 2C, [28]). *crn* null mutants are phenotypically weaker than *clv1-101* null mutants (Fig 2C). However, unlike *clv1-101*, *crn* null mutants were strictly additive with *bam1 bam2 bam3* triple mutants in FMs and *crn-10 bam1 bam2 bam3* plants were identical to *crn* alone (Fig 2C). In addition, *crn-10 bam1 bam2 bam3* displayed a *bam1 bam2 bam3* vegetative SAM phenotype, and lacked the unregulated SAM over-proliferation seen in *clv1-101 bam1 bam2 bam3* plants (Fig 2B) [39]. This observation is consistent with CRN being dispensable for CLV1 mediated regulation of *BAM* expression and signaling *in vivo*.

### Ectopic BAM receptors auto-dampen their own expression in the SAM in *clv1* null mutants

**Fig 3:**
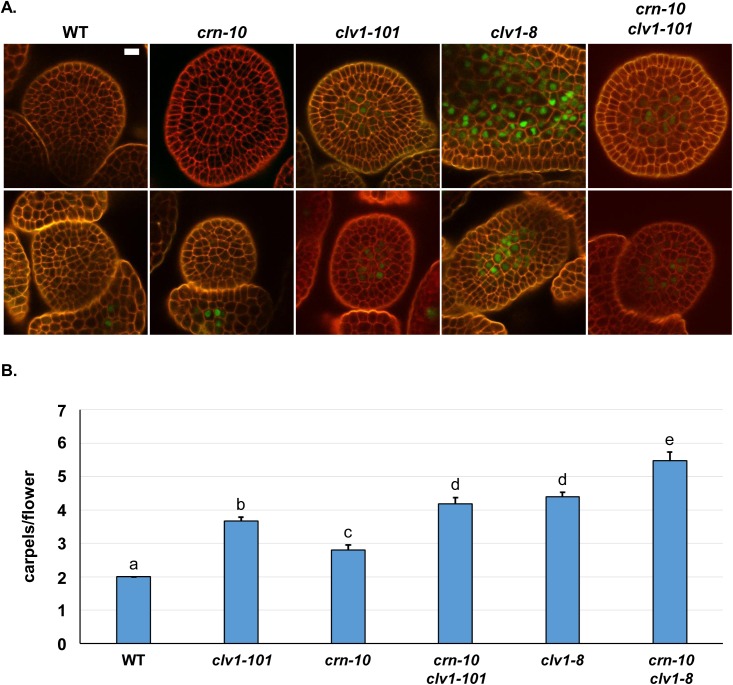
*BAM3* repression is weak in *clv1-101* null mutants. (A) *pBAM3*::*Ypet-N7* signal (green) is not detectable in the center of WT or *crn-10* SAMs (top row) or FMs (bottom row) but is strongly de-repressed in strong *clv1-8* mutant plants which encode an interfering clv1 protein. In contrast, *BAM3* expression is weakly de-repressed in *clv1-101* null mutant plants. This weak de-repression is not due to redundancy with *CRN*, as *crn-10 clv1-101* plants display similar low *BAM3* levels as seen in *clv1-101* alone. Imagining as in Fig 2. White bars, 10 μM. All subpanels are at the same image magnification. Experiment repeated twice. (B) Additive genetic interactions between *crn-10* null mutants and *clv1* null and strong alleles. Y-axis, mean number of carpels per flower for different genotypes. N = 100, experiment repeated twice. Error bars represent the calculated 95% C.I. Letters above bars indicate significance groups as determined by Tukey HSD test/ANOVA.

I previously assessed *BAM3* reporter expression in strong alleles of *clv1* [28], using the *clv1-8* allele in Col-0 which contains a D295N mutation implicated in CLV3p binding [11]. Strong alleles of *clv1* are weakly dominant negative and the molecular basis of this remains unclear [29]. Previous work has suggested strong clv1 mutant receptors could be interfering with CRN [16], or perhaps BAM1 and BAM2 [30]. To test these possibilities I first examined *BAM3* expression in the *clv1-101* null allele in Col-0. *BAM3* reporter expression was considerably lower in L3-L5 cells of *clv1-101* null SAMs compared to both *clv3* null and *clv1-8* strong alleles (Fig 3 and Fig 2A, S3 Fig). In some *clv1-101* null plants, *BAM3* expression was nearly undetectable in the SAM center. Expression of *BAM3* in FMs was more reliably detected in *clv1-101* null plants, but never approached the levels seen in *clv3* null and *clv1-8* strong alleles (Fig 3, Fig 2A). Unlike the low levels of *BAM3* expression in *clv1-101* null plants, *BAM3* reporter expression in *clv2*, *crn* or *rpk2* was not observed (Fig 2A, S3 Fig). Therefore, while *BAM3* expression is de-repressed in the center of the SAM in both null and strong *clv1* alleles, the level of de-repression of *BAM3* is higher in the strong *clv1-8* allele. This result implies that in *clv1-101* null mutants, unknown receptor(s) signaling is still effective at repressing *BAM3* expression. This reduced repression is not due to co-operative receptor function with CRN, as *crn clv1-101* double null mutants contained levels of the *BAM3* reporter equivalent to the *clv1-101* null alone (Fig 3A).

**Fig 4:**
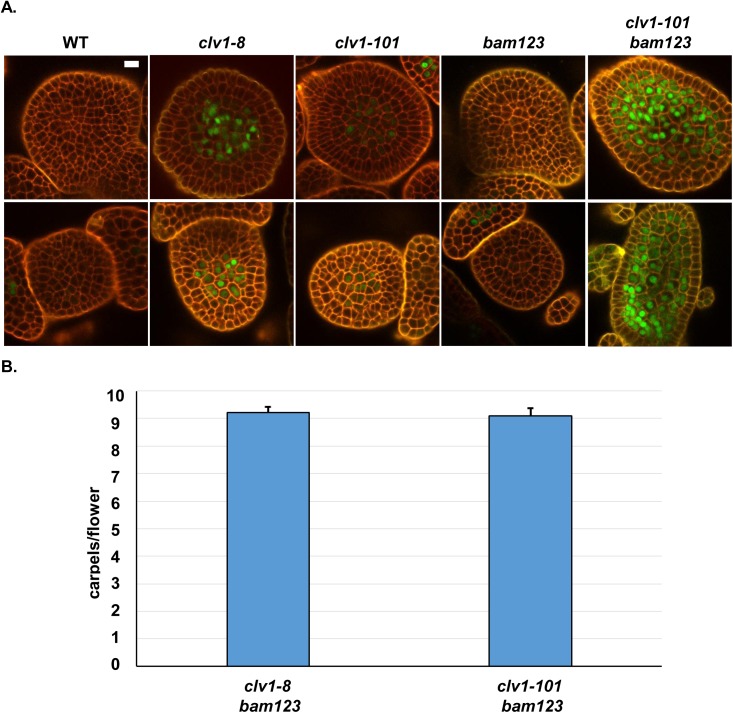
Ectopic BAM receptors feedback to dampen their own expression in the absence of CLV1. (A) Ectopic BAM receptors dampen their own expression in *clv1*-*101* null mutants. In WT or *bam1 bam2 bam3* (*bam123*) triple mutants SAMs (top row), or FMs (bottom row), *BAM3* (green) is effectively repressed by CLV3-CLV1 signaling in the meristem centers. In *clv1-101* null mutant plants, *BAM3* is partially repressed compared to strong *clv1-8* mutant allele plants. Removal of all *BAM* function from *clv1-101* null mutant plants (*clv1-101 b123*) results in de-repression of *BAM3* to levels occasionally higher than *clv1-8* plants, indicating that BAM receptor signaling in *clv1-101* null mutants partially represses *BAM* target genes in the absence of *CLV1*. Imaging as in Fig 2. White bars, 10 μM. All subpanels are at the same image magnification. Experiment repeated 3 times. (B) clv1 strong alleles act via interfering with ectopic BAM receptors. Y-axis, mean number of carpels per flower for different genotypes. N = 80 flowers per genotype, experiment repeated three times. Error bars, represent calculated 95% C.I. Difference not significant at 95% as determined by T-test.

Since BAM receptors are ectopically expressed in *clv1-101* null SAMs and partially compensate for *clv1* they are capable at some level of signaling like CLV1 [28]. I therefore reasoned that ectopic BAM receptors might be dampening their own expression in the center of the SAM in *clv1-101* null mutants. To test this I generated *bam1 bam2 bam3* and *clv1-101 bam1 bam2 bam3* quadruple mutant *pBAM3::Ypet-N7* transgenic lines. Consistent with the previous observation that *bam1 bam2 bam3* are dispensable for CLV1 function [28], *BAM3* was fully repressed in the center of either SAMs or FMs in *bam1 bam2 bam3* mutant plants. In contrast, the weak expression of the *BAM3* reporter in the center of SAMs and FMs in *clv1-101* null plants was greatly enhanced in *clv1-101 bam1 bam2 bam3* quadruple mutants (Fig 4A). This result demonstrates that ectopically expressed BAM receptors in the center of *clv1-101* null mutant SAMs signal to dampen their own expression. The level of *BAM3* reporter de-repression in *clv1-101 bam1 bam2 bam3* plants was comparable to, and occasionally stronger, than in *clv1-8* alleles [28]. Previous work has suggested that clv1 missense receptors could interfere with BAM function [30] or perhaps CRN [16]. To test these hypotheses, I generated *crn-10 clv1-8* double mutants and *clv1-8 bam1 bam2 bam3* mutants. Consistent with the *BAM3* imaging, *crn-10 clv1-8* mutants were additive with respect to carpel number compared to single mutant plants (Fig 3B, S5 Fig), indicating that clv1-8 receptors do not interfere with CLV2/CRN signaling genetically. If clv1-8 receptors were interfering with the function of unknown receptors, and not BAM receptors, then *clv1-8 bam1 bam2 bam3* should be as strong, or stronger, than quadruple null mutants. However, I found that *clv1-8 bam1 bam2 bam3* were comparable to *clv1-101 bam1 bam2 bam3* quadruple null mutants (Fig 4B). These data support the hypothesis that clv1-8, and presumably other strong missense clv1 receptors, function exclusively by interfering with the signaling of ectopic BAM receptors in the SAM. This result is consistent with the *BAM3* reporter imaging demonstrating that *CRN* is dispensable for CLV1 signaling, and also implies that like CLV1, BAM receptors signal *in vivo* in a CLV2/CRN independent manner.

### Functional independence of *WUS*-induced stem cell proliferation and *BAM3* repression

**Fig 5:**
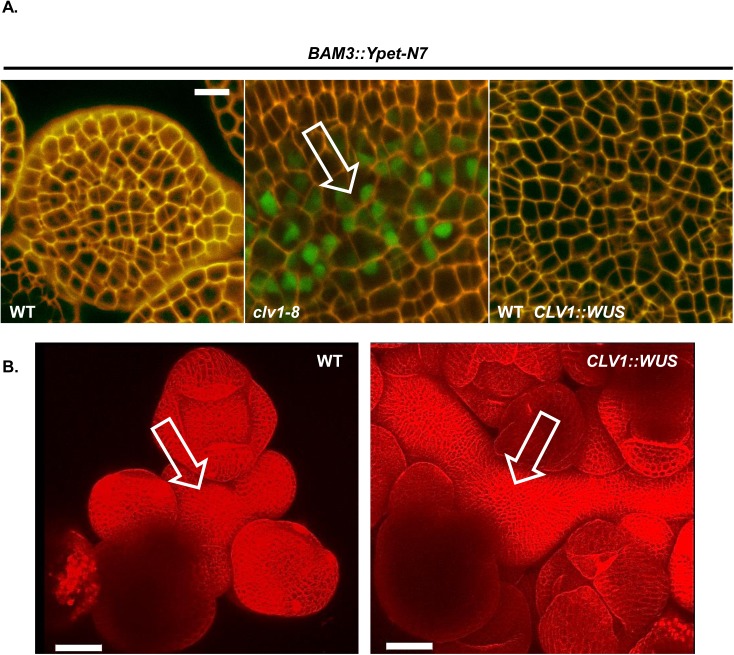
CLV1/CLV3 regulation of BAM expression is separable from *WUS*- mediated stem cell proliferation (A) *WUS*-induced SAM proliferation is separable from *BAM3* repression by CLV1. Wild type (WT) *pBAM3*::*Ypet-N7* plants were imaged and compared to *clv1-8 pBAM3*::*Ypet-N7* and WT *pBAM3*::*Ypet-N7* plants transformed with *pCLV1*::*WUS* transgene. No ectopic BAM3 signal was seen in *pCLV1*::*WUS* lines (N = 10), despite the massive over-proliferation of SAM tissue in some lines (B). White bars, 10 μM. All subpanels are at the same image magnification. B) 3D surface reconstruction of image stack obtained in red channel (propidium iodide, cell wall stain). White bars, 50 μM. All subpanels are at the same image magnification.

CLV1 signaling strongly represses *BAM* expression, but has a considerably weaker effect on *WUS* in physiological ranges of CLV3p [11, 28, 33, 34]. The current model in the field postulates that upregulation of *WUS* drives the excess cell proliferation in *clv* class meristems. I sought to test if *WUS* up-regulation was causally connected to *BAM* de-repression in the SAM of *clv1* mutants. To do this I repeated the experiments in Schoof *et al* [31] and used the *CLV1* promoter to express *WUS* in the SAM of Col-0 *pBAM3::Ypet-N7* plants. In those experiments, ectopic expression of *WUS* using a two-component inducible system drove SAM enlargement and resulted in flowers with extra carpels, phenocopying *clv1* mutants. Of the 36 T1 *CLV1p*::*WUS* lines generated in the Col *pBAM3*::*Ypet-N7* background, only 11 plants displayed increases in SAM size and stem fasciation. Despite having enlarged SAMs reminiscent of *clv1* SAMs (Fig 5B), no de-repression of *BAM3* was observed in the SAM or FMs of any of the enlarged meristem plants examined (N = 10, Fig 5A). This indicates that *WUS*-induced over-proliferation of the stem cell niche is genetically separable from *BAM3* transcriptional regulation in the CLV1 pathway.

## Discussion

**Fig 6:**
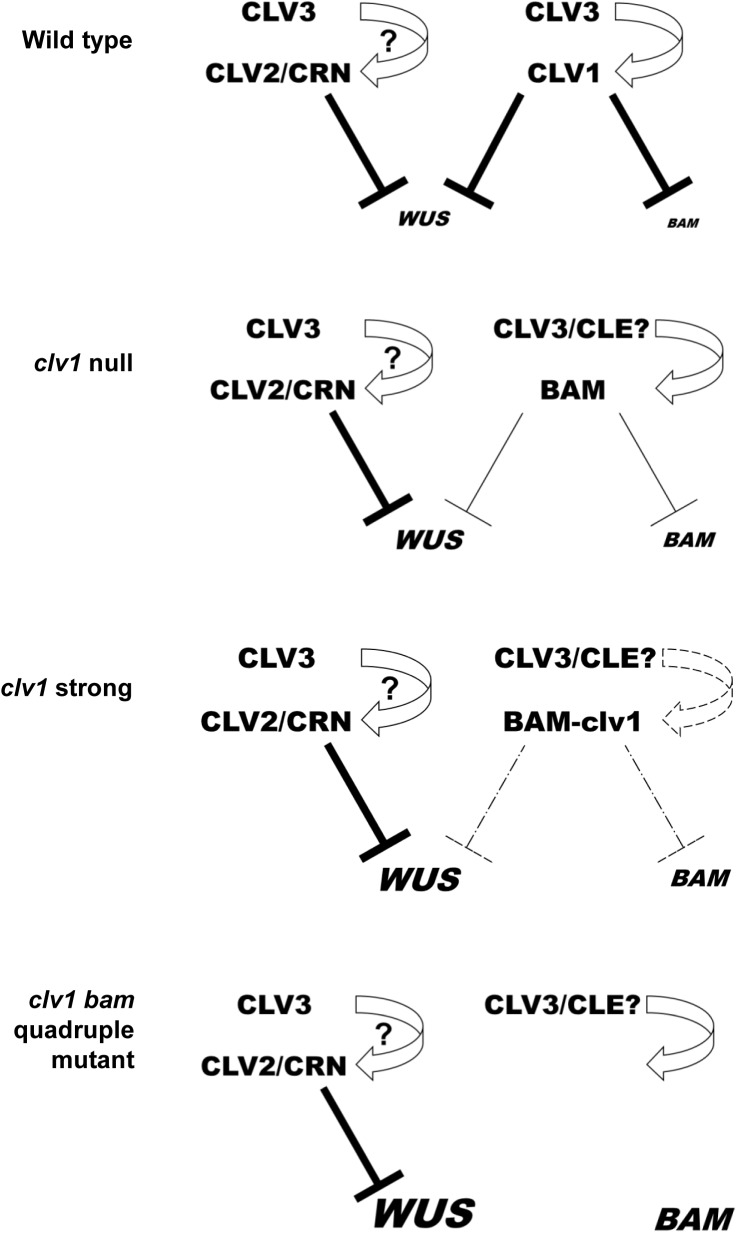
Model for CLV3-receptor signaling circuits *in planta*. Model for CLV3p mediated regulation of *WUS* and *BAM* target genes *in planta*. CRN/CLV2 dimers do not participate in *BAM* regulation which is governed by CLV3-CLV1 signaling. In *clv1* null mutants, ectopic BAM receptors partially replace CLV1 but also dampen their own expression. In *clv1* strong alleles, mutant clv1 receptor interfere with BAM receptor signaling. In *clv1 bam1 bam2 bam3* quadruple receptor mutant plants repression of *BAM* is lifted. The strength of repression is represented by the repression line thickness and target gene expression levels are depicted by font size. Question marks reflect the potential for direct binding CLV3p to CLV2/CRN dimers [23,19] and the existence of postulated CLV3p redundant CLE peptides based on observation that *clv1 bam1 bam2 bam3* quadruple receptors mutants are stronger than *clv3* null mutants. [28].

Despite being cloned nearly 20 years ago, we have little understanding about of how CLV1 signals *in planta* or its relationship to other proposed CLV3p receptors. Here I demonstrate, using *BAM3* repression as a readout, that CLV1 signals to control at least some transcriptional outputs independent of CLV2/CRN and RPK2 but fully dependent on *CLV3* (Fig 6). CLV2/CRN have no effect on *BAM3* reporter expression by themselves, or in combination with CLV1, and genetically do not participate in *BAM* feedback compensation. As such, despite the different receptor mutants having similar qualitative stem cell defects and resistance to CLV3p, CLV1, RPK2 and CLV2/CRN are functionally separate and converge on distinct signaling outputs *in vivo*. This result implies that CLV2/CRN are dispensable for CLV3p mediated perception by CLV1, and that putative CLV2/CRN/CLV1 complexes seen in tobacco overexpression studies are dispensable for CLV1 signaling *in vivo*. This is consistent with previous data showing that CLV1 traffics from the plasma membrane to the lytic vacuole in response to CLV3p in a *CLV2*-independent manner [10], and consistent with additive genetic interactions with *clv2*, *crn* and *clv1* [16]. As such, every readout for CLV1 function would suggest that CLV2/CRN are dispensable for CLV3p perception and signaling *in vivo*. Previously it was suggested that strong clv1 receptors could interfere with CLV2/CRN function *in vivo* [16], however I found that there is a significant enhancement of the strong *clv1-8* allele in *clv1-8 crn-10* double mutants. Genetic analysis and *BAM3* repression analysis suggests that the strength of the clv1-8 mutant receptor can be accounted for solely by interfering with ectopic BAM receptors in SAM, supporting previous studies [30].

Despite their independence from CLV1, *clv2* and *crn* mutants are resistant to ectopic CLE peptide-mediated SAM or RAM termination in several species of plants, and have not been identified in genetic screens for other peptide responses to date. This suggests that there is either a tight relationship between CLV2/CRN and CLV3/CLE ligand function, or that CLV2/CRN impact a developmental process which superficially resembles CLV3p function. Interestingly, while *CLV2* and *CRN* mutants display resistance to CLV3/CLE peptide induced root termination [16, 40], they do not display conspicuous root growth or patterning defects in the absence of peptide ligand [16]. I previously demonstrated that mutants that do not function in the *CLV3* pathway, but have a higher rates of SAM cell proliferation and an expanded SAM, display resistance to CLE peptide mediated SAM termination [28]. This suggests that CLE peptide resistance *per se* is insufficient to determine gene function in CLV3p signaling or perception. Based on this, it is formally possible that CLV2/CRN do not function in CLE mediated perception *in vivo*, as has been suggested by peptide binding assays [23]. The function of CLV2/CRN in CLE mediated signaling, if any, remains enigmatic, but current data demonstrates that CLV1 ligand perception and binding, stability, endomembrane trafficking, and signaling are all independent of CLV2/CRN *in planta*.

*clv1* null mutants are phenotypically weak, despite having ectopic *BAM* expression in the SAM center. However, like CLV1, BAM receptors also repress *BAM* expression in the center of the SAM (Fig 6). As such, *clv1* null mutant SAMs contain low levels of *BAM* expression, potentially explaining why ectopic *BAM* expression is not sufficient to fully compensate for *clv1*. In the organizing center, CLV1 is proposed to also repress *WUS* expression. Despite this, *CLV1* and *WUS* expression is largely co-incident and expression of either *CLV1* or *CRN*/*CLV2* in *WUS*-expressing cells is sufficient to account for all functions in stem cell regulation. WUS induced SAM over-proliferation can be uncoupled from CLV1-mediated *BAM* repression. As such, *clv1* and *clv3* SAMs are functionally different from WUS-induced *clv*-like SAMs at some level. WUS itself has been proposed to repress *CLV1* [33], but the significance of this is unclear due to the co-incident expression patterns of both genes, the lack of de-repression of *BAM3* in enlarged SAMs ectopically expressing *WUS*, and the fact that uncoupling *CLV1* expression from the native *CLV1* promoter in SAM cells has no phenotypic consequence other than to complement *clv1* null mutants [10, 28–30, 39]. In addition, CLV1 mediated repression of *WUS* is quantitatively different from that for *BAM* receptors. In the center of wild type SAMs *BAM* gene expression is nearly undetectable and becomes robustly detectable in *clv1* or *clv3* mutants. In contrast, *WUS* is robustly detected in wild type SAMs [28, 31]. Overexpression of *CLV3* represses *WUS*, however plants expressing up to 300 fold more *CLV3* are wild type in appearance [34]. Thus, at endogenous levels CLV3p strongly suppresses *BAM* expression in a *CLV1*-dependent manner, but has less of an effect on *WUS*, which requires considerably higher and potentially non-physiological levels of CLV3p for full repression. Understanding the regulation of *BAM* expression by CLV1 could lead to new insights into this signaling pathway.

## Materials and methods

### Plant growth, genetics and selection

Plant growth and transgenic plant selection was performed as described in [28]. All *clv1*, *bam1*, *bam2*, *bam3* and *clv2* alleles are in the isogenic Col-0 background and have been characterized previously. Genotyping of plants from crosses were performed using appropriate primers selecting for mutant alleles or T-DNA insertions [28, 35]. Carpel counts were performed as described with the exception of comparisons using *crn-10* or *clv2*. In these lines early termination of the SAM and the cessation of flower production was observed after 5 flowers on average as noted before for *clv2* mutations in the Col-0 background [35]. Therefore, I counted flowers 6 and beyond for all comparisons using *clv2* or *crn-10* alleles for all genotypes in those experiments. The transient termination phenotype of *crn-10* was not altered by mutations in *clv1*, *clv3* or in *bam1 bam2 bam3* triple null mutants. In *bam1 bam2 bam3 clv1* plants stems elongation is highly distorted as is SAM production as described in [28], making inferences about floral primordia order difficult. As such, all flowers were counted for comparisons between quadruple mutants.

### Confocal imaging

Confocal imaging was performed using an inverted Zeiss 710 confocal. Briefly, an inverter adaptor (LSM Tech, Etters, PA, USA) was used to allow upright imaging of shoot meristems when attached to the Zeiss 710. Details on the configuration of the inverter are available on request and will be published elsewhere. Meristem staging, dissection and mounting were performed as described in [28], with each presented photo being a mean of eight scan cycles. For each experiment a minimum of 6 meristems were imaged and all imaging experiments were repeated with different plant populations two to four times. Imaging settings for the Ypet channel were kept constant across all experiments, but the gain on the red channel for propidium iodide was altered to account for staining differences as necessary. Image settings were calibrated to capture the dynamic linear range in most plants. At these image settings YFP signal in *clv1 bam1 bam2 bam3* quadruple mutant SAMs was occasionally saturating in some nuclei (S2 Fig) but no signal was detectable in wild type, *clv2*, *crn* or *bam1 bam2 bam3* SAM at these settings (S2 Fig, Figs 2–5).

### Generation of binary vectors

The *CRN* promoter binary vectors used were described in [17]. For the *CLV2* promoter binary, 1250 bp and 588 bp of the 5' and 3' promoter and UTR regions were amplified from Col-0 and fused together using recombinant PCR to introduce a unique BamH1 site and cloned into pBJ shuttle vector. A Gateway cloning cassette was inserted into the BamH1 site and the entire promoter cassette was transferred into the *pMOA33* binary vector as a Not1 fragment [41]. Col-0 *CLV2* CDS was amplified using primers that allowed cloning into the *pENTRD* (Invitrogen) vector and fused with an in frame MYC epitope to the C-terminus. This was then recombined into either *pMOA33 CLV2p* or *pMOA33 WUSp*. The *pMOA33 WUSp* vector was described as in [28]. For the generation of *pMOA33 CLV1p*::*WUS*, a *pENTRD WUS* CDS clone was recombined into the *pMOA33 CLV1p* vector used previously [10, 28].

### Creation of the *crn-10* and *rpk2-cr* null allele mutants

The *pCUT* vector system was used to generate the *crn-10* allele and *rpk2-cr* allele used here [36]. Briefly, this vector series co-expresses nuclear targeted Cas9 from the *UBQUITIN10* promoter and gRNAs from the *U6* promoter. A 20 base pair gRNA target site (bold), including upstream *G* and downstream PAM (underlined) was selected g**aagcaaagaagaagaagaaa**tgg near the initiator methionine codon in the *CRN* genomic signal sequence encoding region. This target site was used to generate a gRNA that was cloned into *pCUT3* as described in [36]. Kanamycin resistant plants were selected in the T1. In a couple of T1 plants, branches were observed with flowers which all contained elevated levels of carpels relative to wildtype. Seeds were collected specifically from one of these branches and sequencing in the next generation revealed that these branches arose as somatic bi-allelic sectors containing either an A or T insertion (bold) at the same location downstream of the initiator ATG (uppercase) (ATGaagcaaagaagaagaag**T**aaatgg) leading to equivalent truncated CRN proteins with only 7 amino acids remaining in the signal sequence of CRN (MKQRRRRKWMstop). Plants with the homozygous T insertion event were selected as this provides resistance to Hph1 digestion using the dCAPs primers CRN-10 F gtagaagcagcaatgaagcaaagaagaaggtg and CRN-10 R gttgaagttgtggataagtg [42], and segregated away from the *pCUT3* transgene. Complementation analysis was performed using vectors described in [17]. For *rpk2*-cr mutant creation, a tandem array of two different *U6* promoter gRNA cassettes targeting the *RPK2* CDS were gene synthesized by Invitrogen and cloned into *pCUT3* as described in [36]. The *RPK2* gRNA target sites chosen were aagattactgctcctggtt**tgg** and tcatggctcttaacattag**tgg**, with the PAM sequence in bold. This vector was transformed directly into the Col-0 *pBAM3*::*Ypet-N7* line. In the T2 generation from a select T1 line, multiple plants displaying an *rpk2* phenotype were identified based on male sterility and extra carpels [18, 43]. Imaging was performed on 10 plants with an *rpk2* phenotype. One line lacking the *pCUT3* vector was identified by PCR and backcrossed to Col-0 to maintain as a heterozygous owing to the male sterility of *rpk2* mutants [43]. This line was then imaged again in the next generation to confirm *BAM3* expression patterns. This line, termed *rpk2-cr*, contains a +A insertion between nucleotides 229 and 230 and an additional +T insertion between nucleotides 279 and 290 in the *RPK2* CDS. This results in the production of an RPK2 protein that contains a stop codon directly after amino acid 76 (serine76) downstream of the signal sequence resulting in the deletion of all LRR repeats and downstream transmembrane, juxtamembrane and kinase domains [43].

## Supporting information

S1 Fig Complementation of the *crn-10* null mutant by *CRN* and *CRN* variants. (A) *crn-10* is fully complemented by *CRN*, kinase enzymatically dead *CRN* (*CRN*_*K146E*_) and serine 156 substitution mutants (CRN_S156A_ and CRN_S156D_). Full complementation was designated as every flower containing two carpels as in wild plants. (B) *CRN* is expressed in the SAM center and center of developing FMs. *crn-10* mutant plant complemented with *pCRN*::*CRN-2xGFP* transgene. Images of the meristem center, determined by appearance of chloroplasts in L3 [10]. CRN-2xGFP signal appears at presumptive PM and signal was not seen in tonoplast or perinuclear ER. CRN-2xGFP fully complemented *crn-10* in all lines examined (n = 20) like 2x mCherry, however, signal in CRN-2xmCherry plants was below detection limit in the SAM most lines likely owing to lower intrinsic brightness of mCherry.
(TIF)

S2 Fig Calibration of *pBAM3* Ypet-N7 signal for imaging. At the calibrated imaging settings (3.5% 514 nm laser power, pinhole 121 μM, scans averaged) in this study saturating levels of nuclear Ypet signal are seen in some *clv1 bam1 bam2 bam3* nuclei (yellow arrow) but signal is undetectable in wild type plants at same setting.
(TIF)

S3 Fig *pBAM3*::*Ypet-N7* expression differences *clv1-8* and *clv1-101* mutants. Detail from Fig 4. Note that images were brightened and enlarged equally in PowerPoint relative to Fig 4 in order to magnify detail and illustrate the intensity differences between *pBAM3*::*Ypet-N7* signal (green nuclei) in *clv1-8* and *clv1-101*.
(TIF)

S4 Fig Lack of *BAM3* is expression is SAMs is not due to silencing of *pBAM3*::*Ypet-N7*. Imaging of *bam1 bam2 bam3 pBAM3*::*Ypet-N7* (green) plants as an example of genotypes in which *BAM3* signal is below detection in the SAM center (left, white bars = 10 μM), but still expressed in developing vasculature/phloem below the SAM proper (right, green arrows, white bars = 20 μM).
(TIF)

S5 Fig Carpels per flowers in *clv1-8* and *crn-10 clv1-8* double mutants. Distribution of flowers with specific carpel numbers. *clv1-8*, blue bars; *clv1-8 crn-10* double, orange bars. Data from Fig 3B. N = 100, experiment repeated twice. Y-axis, total number of flowers. X-axis, carpel number per flower.
(TIF)
