# Supplementary figures and images for: CLAVATA1 controls distinct signaling outputs that buffer shoot stem cell proliferation through a two-step transcriptional compensation loop
Source: PLoS Genet. 2017 Mar 29;13(3):e1006681. doi: 10.1371/journal.pgen.1006681 (PMC5371295; doi:10.1371/journal.pgen.1006681)

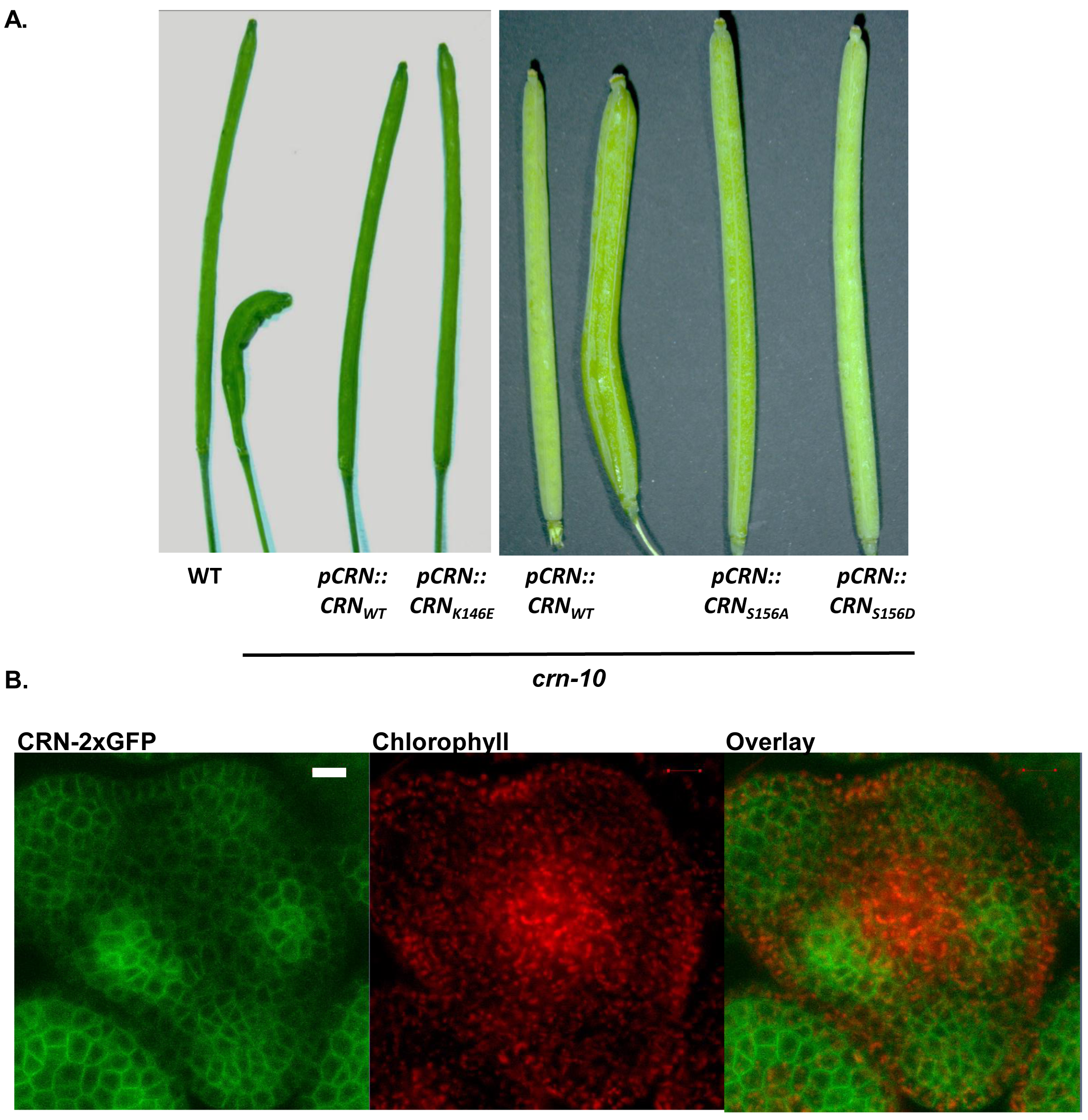

Supplement: S1 Fig — (A) crn-10 is fully complemented by CRN, kinase enzymatically dead CRN (CRNK146E) and serine 156 substitution mutants (CRNS156A and CRNS156D). Full complementation was designated as every flower containing two carpels as in wild plants. (B) CRN is expressed in the SAM center and center of developing FMs. crn-10 mutant plant complemented with pCRN::CRN-2xGFP transgene. Images of the meristem center, determined by appearance of chloroplasts in L3 [10]. CRN-2xGFP signal appears at presumptive PM and signal was not seen in tonoplast or perinuclear ER. CRN-2xGFP fully complemented crn-10 in all lines examined (n = 20) like 2x mCherry, however, signal in CRN-2xmCherry plants was below detection limit in the SAM most lines likely owing to lower intrinsic brightness of mCherry. (TIF) [file pgen.1006681.s001.tif]

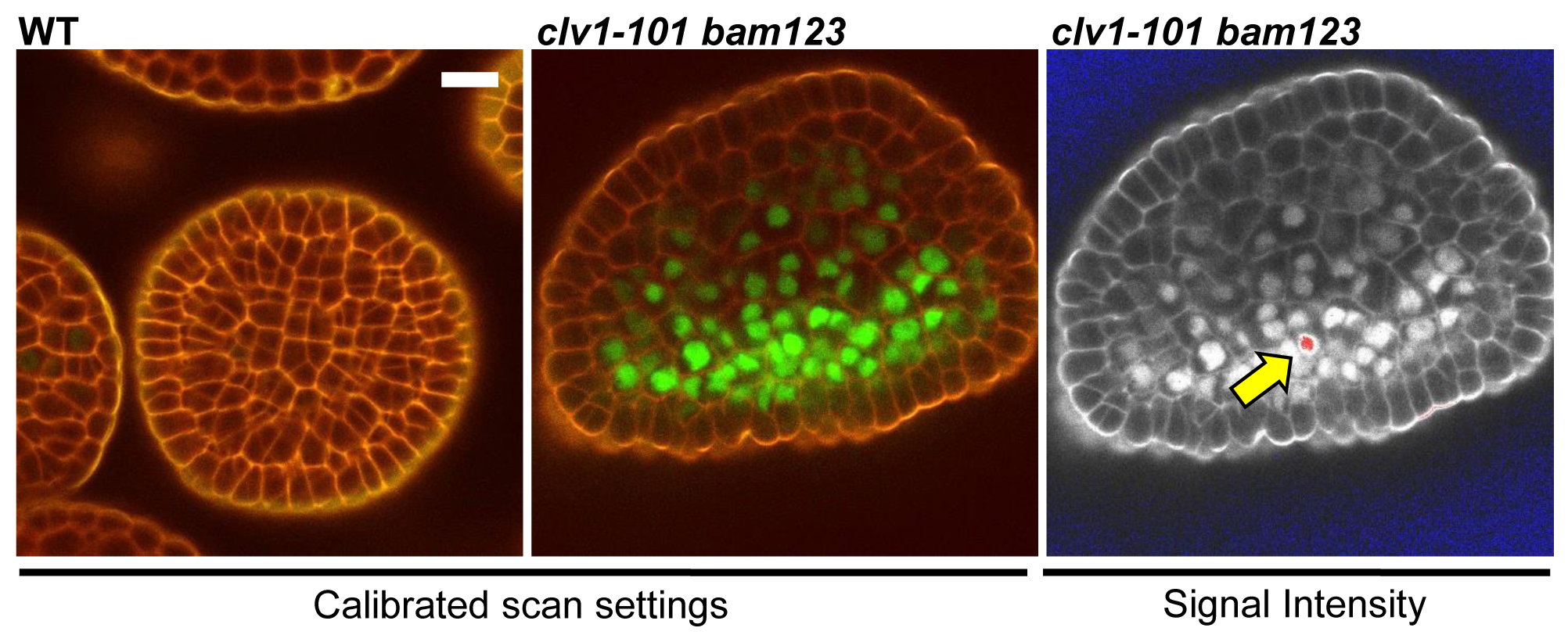

Supplement: S2 Fig — At the calibrated imaging settings (3.5% 514 nm laser power, pinhole 121 μM, scans averaged) in this study saturating levels of nuclear Ypet signal are seen in some clv1 bam1 bam2 bam3 nuclei (yellow arrow) but signal is undetectable in wild type plants at same setting. (TIF) [file pgen.1006681.s002.tif]

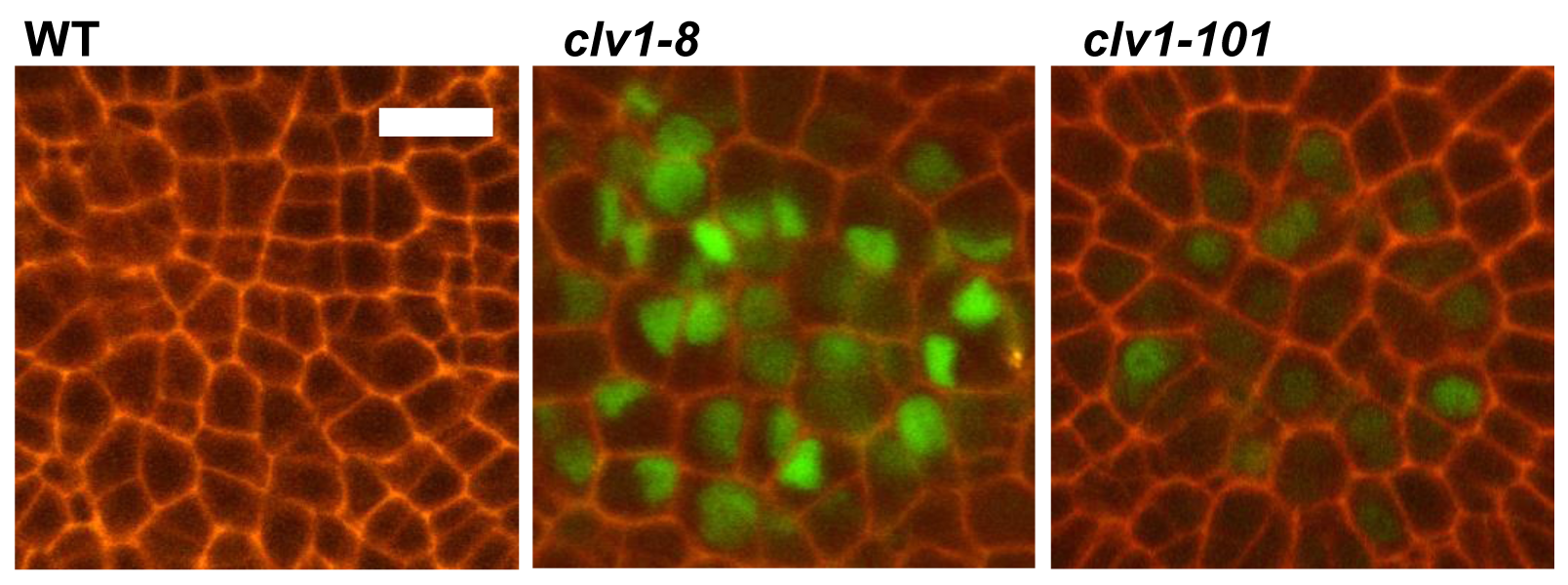

Supplement: S3 Fig — Detail from Fig 4. Note that images were brightened and enlarged equally in PowerPoint relative to Fig 4 in order to magnify detail and illustrate the intensity differences between pBAM3::Ypet-N7 signal (green nuclei) in clv1-8 and clv1-101. (TIF) [file pgen.1006681.s003.tif]

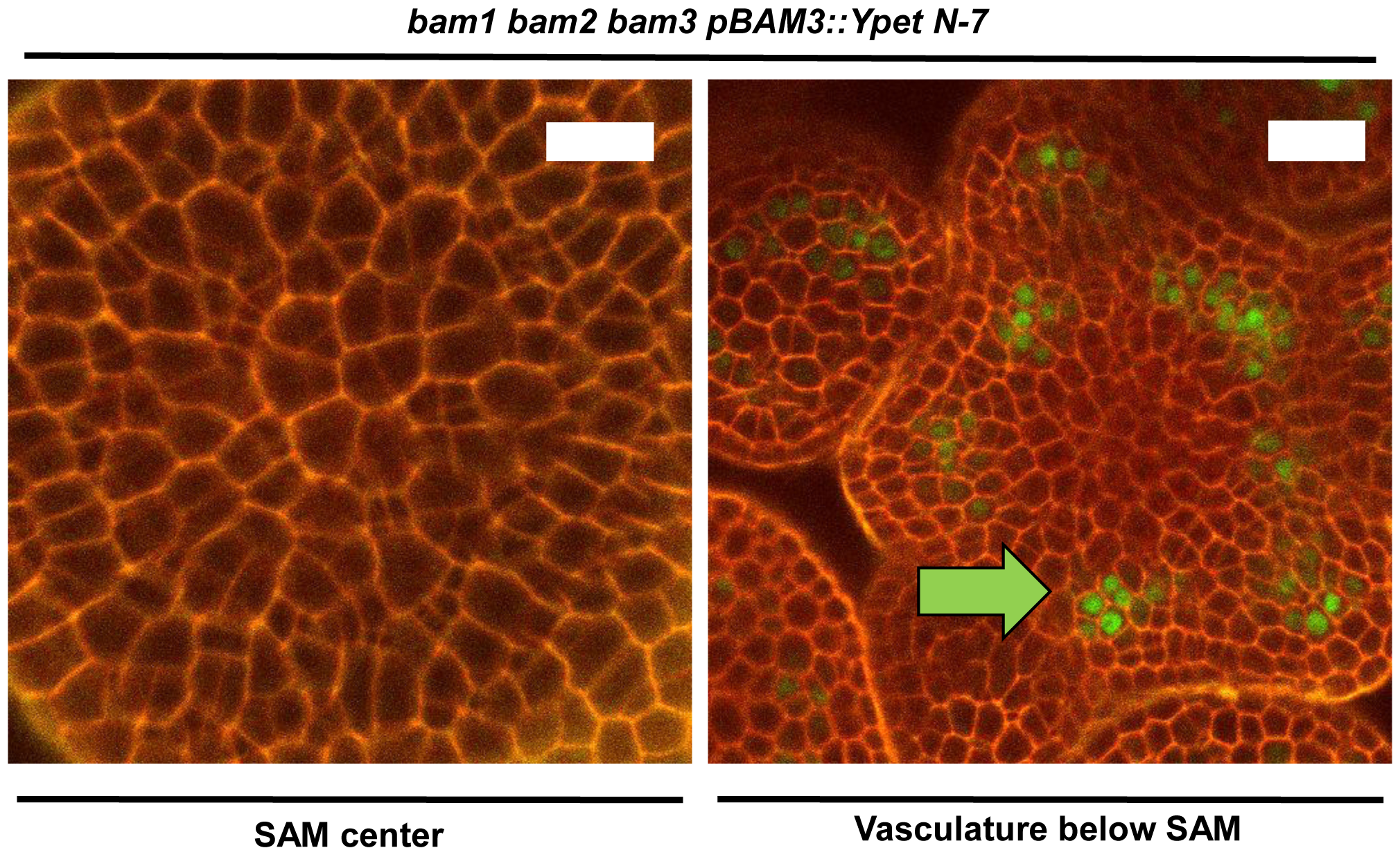

Supplement: S4 Fig — Imaging of bam1 bam2 bam3 pBAM3::Ypet-N7 (green) plants as an example of genotypes in which BAM3 signal is below detection in the SAM center (left, white bars = 10 μM), but still expressed in developing vasculature/phloem below the SAM proper (right, green arrows, white bars = 20 μM). (TIF) [file pgen.1006681.s004.tif]

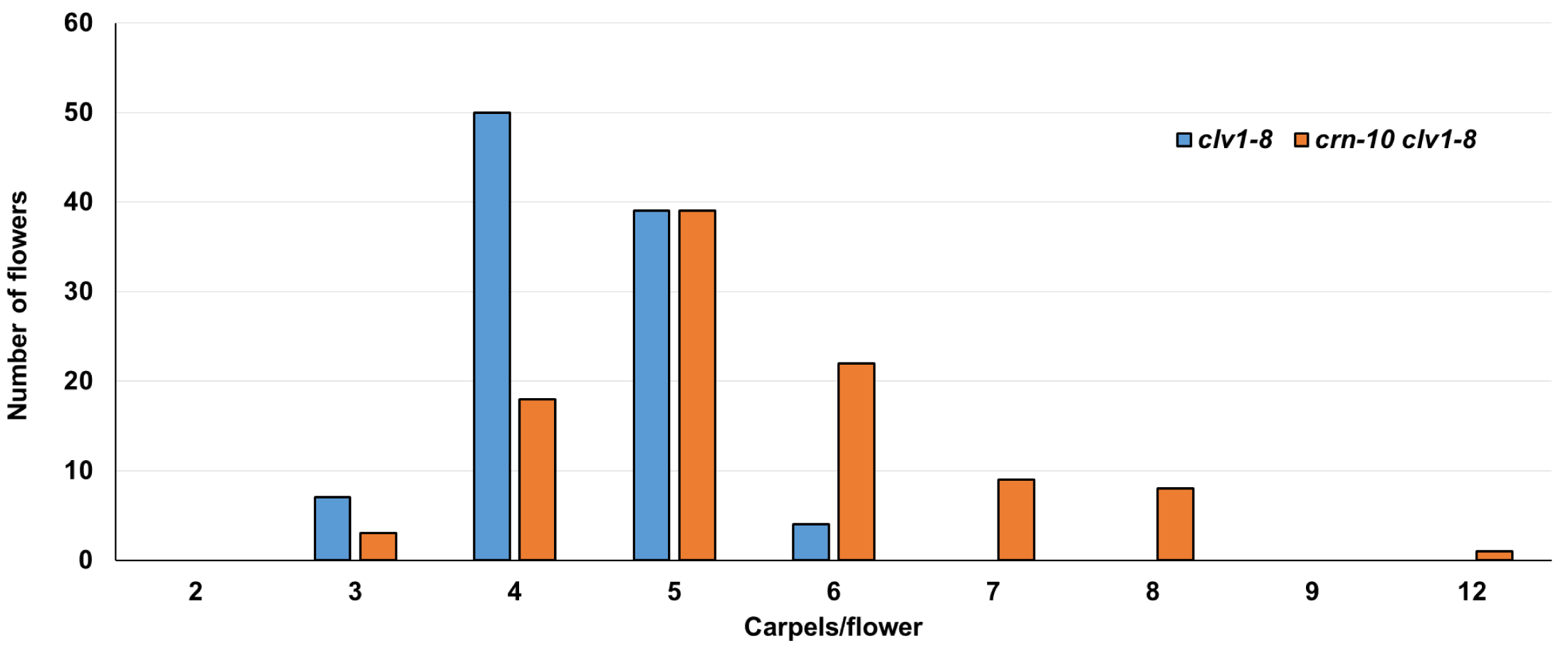

Supplement: S5 Fig — Distribution of flowers with specific carpel numbers. clv1-8, blue bars; clv1-8 crn-10 double, orange bars. Data from Fig 3B. N = 100, experiment repeated twice. Y-axis, total number of flowers. X-axis, carpel number per flower. (TIF) [file pgen.1006681.s005.tif]
